# Social inequality in food consumption between 2008 and 2019 in Brazil

**DOI:** 10.1017/S1368980021002950

**Published:** 2022-02

**Authors:** Barbara Virginia Caixeta Crepaldi, Letícia Martins Okada, Fernanda Rauber, Renata Bertazzi Levy, Catarina Machado Azeredo

**Affiliations:** 1 Programa de pós-graduação em Ciências da Saúde, Faculdade de Medicina, Universidade Federal de Uberlândia, Av Pará, 1720, Bloco 2 U, Umuarama, Uberlandia, MG 38405-320, Brasil; 2 Departamento de Nutrição, Faculdade de Saúde Pública, Universidade de São Paulo, São Paulo, Brasil; 3 Departamento de Medicina Preventiva, Faculdade de Medicina, Universidade de São Paulo, São Paulo, Brasil

**Keywords:** Inequality, Education, Food consumption, Foods, Surveillance

## Abstract

**Objective:**

To analyse the trend of social inequality in food consumption among Brazilians from 2008 to 2019.

**Design:**

Time series analyses using cross-sectional annual data from the Telephone Surveillance System (VIGITEL 2008–2019). Food consumption was evaluated through: (1) consumption of five or more portions of fruits and vegetables in ≥5 d/week; (2) consumption of beans in ≥5 d/week and (3) consumption of soft drinks or artificial juices in ≥5 d/week. Absolute inequality was assessed by the slope index of inequality (SII) and relative inequality by the concentration index (CIX). SII and CIX positive values indicate higher prevalence among more educated citizens and negative among less educated ones. Time trend was assessed by linear regression using weighted least squares.

**Setting:**

26 Brazilian state capitals and the Federal District.

**Participants:**

621 689 individuals ≥18 years.

**Results:**

Fruits and vegetable consumption was more prevalent among the more educated citizens, while beans were mostly consumed by the less educated, and soft drinks or artificial juices was more prevalent among individuals with intermediate education. The highest absolute inequality was found for beans (SII_2019_ -25·9). In 12 years, the absolute inequality increased for fruit and vegetable consumption (from SII_2008_ 12·8 to SII_2019_ 16·2), remained for beans (SII_2008_ -23·1 to SII_2019_ -25·9) and reduced for soft drinks or artificial juices (SII_2008_ 8·7 to SII_2019_ 0·4). Relative inequality was low and constant.

**Conclusion:**

Despite the advances reducing inequalities in soft drinks or artificial juice consumption, the increase in the social gap for adequate consumption of fruits and vegetables is troublesome.

Adherence to a healthy diet throughout life contributes to the prevention of malnutrition, chronic noncommunicable diseases (NCD) and deaths^(1)^. The higher consumption of fruits, vegetables and legumes, such as beans, is associated with lower total mortality risk^(2)^. In contrast, the increased consumption of ultra-processed foods, for example, soft drinks and sweetened beverages, is linked to a higher risk of NCD and all-cause mortality^(3)^.

A healthy diet is influenced by socio-economic factors^(1)^. In low- and middle-income countries, increased access to high-quality and expensive nutritional food in addition to energy-rich and low-cost foods has been associated with high socio-economic status. In Brazil, unhealthy eating patterns have already been demonstrated in individuals with low socio-economic status^(4,5)^.

Brazil has significant economic inequality, in addition to ethnic and gender disparities^(6)^, and poverty reaching mainly women and Black or Brown population^(7)^. A review conducted with data from Brazilian national surveys brought to light that individuals with higher income and higher education consume more fruits and vegetables, less traditional Brazilian foods, such as beans, and intake more ultra-processed food, such as soft drinks and/or artificial juices^(4)^. Descriptive outcomes based on sex and race/skin colour indicated that women and White individuals had a higher consumption of fruits and vegetables. Although men consumed more beans, they ate more frequently foods associated with a higher NCD risk, such as fat meats, whole milk, soft drinks and/or artificial juices. Elevated consumption of beans and other food markers for NCD also correlated with Brown and Black skin colours^(4)^.

A slight increase in the proportion of Brazilians achieving the recommended consumption of fruits and vegetables was identified in Brazil between 2008 and 2016, according to the WHO recommendation^(1)^; however, this consumption remained higher among women and citizens with higher schooling levels^(8)^. Between 2006 and 2008, there was a reduction in the regular consume of beans and low consumption was identified among individuals with higher schooling levels, women and White individuals^(9)^. From 2007 to 2016, a reduction was observed in the regular consumption of sweetened drinks, mainly among more educated people and men^(10)^. These previous studies described food consumption according to socio-demographic characteristics without analysing inequality measures and their tendency. They included isolated food indicators, which hampered the understanding of how social inequality would affect healthy and unhealthy food markers more broadly. Moreover, recent data were not shown in their time series. Hence, social inequalities magnitude and their trend in the consumption of food markers for each sex and race/colour remain uncertain.

Therefore, the continuous monitoring of health inequalities is pivotal to record trends and provide relevant information to civil society and governmental authorities^(6)^, thus tackling income, education and gender inequalities within and between countries^(11)^. In light of the above, our objective was to evaluate social inequality trends in the recommended consumption of fruits and vegetables, regular consumption of beans, soft drinks or artificial juices among individuals dwelling in Brazil from 2008 to 2019.

## Methods

### Sampling and data source of the study population

This study used cross-sectional annual data from the Brazilian Surveillance of Risk and Protective Factors for Chronic Diseases through Telephone Interviews (VIGITEL) performed by the Brazilian Ministry of Health from 2008 to 2019. VIGITEL is a cross-sectional monitoring system for the frequency and distribution of the main NCD determinants in individuals aged 18 years or older, residing in households with a fixed telephone line in Brazilian capitals and the Federal District^(12)^.

The sampling process consisted of selecting one resident from each household, after drawing telephone lines by city, stratified according to the region or telephone lines prefix, and through the zip code after the 2012 edition. A final sample weight was assigned to each interviewee to minimise possible sampling biases derived from the partial populational coverage of the fixed telephone system and the difference in the probability of each individual being selected for the study. The final sample weight considered the inverse of the number of telephone lines, the number of adults living in the household of each interviewee and the socio-demographic composition (sex, age range and education level). To adjust the socio-demographic distribution of the VIGITEL sample to the distribution of the adult population in each city, the 2000 demographic census provided by the Brazilian Institute of Geography and Statistics – IBGE was applied for 2008–2011 VIGITEL surveys. From 2012 VIGITEL surveys, in view of the availability of inter-census projections on the socio-demographic distribution of the total adult population in each city, the adjustment was made considering the 2000 and 2010 demographic censuses and their mean annual variation (geometric rate) in the inter-census period. This final weight attributed to each interviewed individual enables the statistical inference of VIGITEL results for the population of individuals aged 18 years or older in each city with and without fixed telephone line and each year of survey edition^(12)^.

Data were acquired from 621 689 individuals interviewed between 2008 and 2019. Regarding the analysis of beans’ regular consumption, 569 294 individuals were included since no data were available for 2018.

Individuals who were not aware of or were not willing to inform their education level (*n* 7123 individuals; 1·2 % of the initial sample) had their data imputed by VIGITEL, using the most frequently observed value based on age and sex^(13)^. To perform the sub-analysis of skin colour and race, those who declared themselves Yellow (*n* 9200; 1·5 % of the initial sample) and Indigenous (*n* 6514; 1·1 % of the initial sample) were excluded due to low representation, which limits the power to detect significant differences within the group. In this sub-analysis, we also excluded all individuals with missing information about their skin colour/race (e.g. who were not aware or did not want to inform) (*n* 33 658; 5·4 % of the initial sample), totalling 572 317 participants included.

### Variables of interest

#### Food consumption: dependent variables

An evaluation of food consumption was performed for the following items: (1) fruits/natural fruit juice and vegetables, (2) beans (healthy eating patterns markers), as well as (3) soft drinks or artificial juices (unhealthy eating patterns marker). The questions format was: ‘How many days a week do you usually eat (or drink) (food or drink)? (1–2 days/week, 3–4 days/week, 5–6 days/week, every day, rarely or never)’. Additional questions were performed to acquire the marker of recommended consumption of fruits and vegetables. Regarding fruits/natural fruit juices, questions were asked about the daily frequency in which they contemplated the options: 1, 2, 3 or more times (for fruits) or glasses (for fruit juice). For vegetables, the questions included raw and cooked options with some examples, such as lettuce, tomato, kale, carrots, chayote, eggplant, zucchini, but not potatoes, cassava nor yams, and the daily frequency included the options: at lunch, at dinner, or lunch and dinner.

The consumption of a fruit or a glass of fruit juice was considered equivalent to one serving, limiting to three the maximum number of daily servings computed for fruits, with the possibility of including a maximum of one glass of fruit juice as a fruit portion. Similarly, the consumption of a vegetable in a meal was also considered equivalent to one portion, limiting the maximum number of daily portions to four, a situation observed among individuals who reported consumption of raw and cooked vegetables both at lunch and dinner. We assumed the estimated intake of five or more portions of fruits and vegetables per day, at least 5 d a week, as daily consumption of 400 g/d (i.e. five portions). These values are recommended by the WHO^(1)^ and in line with the definition adopted by VIGITEL^(12)^. Consumption of beans and soft drinks or artificial juices was categorised as regular when the frequency of consumption was equal to 5 or more days of the week, regardless of the quantity and type, also following the definition adopted by VIGITEL^(12)^.

#### Equity stratifiers: independent variables

Food consumption was described according to years of schooling (presented in four categories: 0–3 years; 4–8 years; 9–11 years; ≥ 12 years), sex (male; female) and skin colour/race (White; Black/Brown).

### Complex measures of inequality

Social inequality was estimated for schooling (educational inequality) by complex inequality measures, such as the slope index of inequality (SII) for absolute inequality and the concentration index (CIX) for relative inequality^(14)^. The SII assesses the absolute difference in a health indicator between the least favoured groups (0–3 years of study) and the most favoured groups (≥ 12 study years). CIX presents the relative difference among them. SII and CIX results, stratified by sex and skin colour/race, considered all educational levels of the population and were calculated based on schooling levels.

### Statistical analysis

Socio-demographic characteristics and food consumption prevalence in the samples (2008–2019) were expressed as either means or frequencies. SII was estimated through logistic regression, a more appropriate analysis of prevalence indicators, while CIX was calculated without corrections^(15)^. Outcomes found for SII and CIX were multiplied by 100, ranging from -100 and +100, to ease graphs comprehension. Results equal to zero represent a total equality situation, while ± 100 results express total inequality. Negative values indicate that the health indicator prevalence is more elevated in less educated groups, while positive values underline a higher health indicator prevalence in the more educated group. CIX results inferior to -20 or superior to +20 indicate an expressive relative inequality^(14)^.

SII and absolute CIX temporal trends for each food consumption indicator were analysed through linear regressions using least squares weighted by variance, based on the mean value and the standard deviation of SII and CIX for each year. Temporal trends with a *P*-value <0·05 were considered statistically significant. To estimate the lines in the graphical representation of temporal evolution, predicted values of SII and CIX were obtained by the Prais–Winsten method with Durbin–Watson autocorrelation and adjusted for the standard error.

An equiplot was generated to present the food consumption inequalities according to schooling levels for each year (www.equidade.org/equiplot). Statistical analyses and graphs plotting were performed using STATA/se software version 16 (StataCorp. LLC), considering the VIGITEL sample design for descriptive analysis (Stata survey prefix command) and the sample weights when estimating SII and CIX measurements.

## Results

Individuals presented a similar distribution profile for age, sex and skin colour between 2008 and 2019. The mean age among participants was about 41 years old. In 2019, most individuals were female and Black/Brown, with a frequency equal to 54·0 % and 50·6 %, respectively. An expressive increase in populational schooling, from 21·6 % in 2008 to 32·8 % in 2019, was identified mainly among individuals with 12 or more years of study. The prevalence of individuals who reported the recommended consumption of fruits and vegetables (≥5 portions/d in ≥5 d/week) was minimal and presented a slight increase over the period (from 20·0 % in 2008 to 22·9 % in 2019). In contrast, regular consumption of beans (≥5 d/week) was noticed in about half of the population during the study period, but a propensity to frequency reduction is noteworthy, varying from 65·6 % in 2008 to 59·7 % in 2019. The frequency of individuals who presented regular consumption of soft drinks or artificial juices (≥5 d/week) decreased expressively (from 26·4 % in 2008 to 15·0 % in 2019) (Table 1).

**Table 1:** Socio-demographic characteristics, consumption prevalence of fruits and vegetables, beans and soft drinks or artificial juices in Brazil, VIGITEL 2008–2019

Characteristics	Survey year											
	2008	2009	2010	2011	2012	2013	2014	2015	2016	2017	2018	2019
	%	%	%	%	%	%	%	%	%	%	%	%
Individuals												
*n*	54 353	54 367	54 339	54 144	45 448	52 929	40 853	54 174	53 210	53 034	52 395	52 443
Mean age in years	40·5	40·7	40·9	41·1	41·3	41·6	41·7	42·0	42·2	42·4	42·6	42·7
Years of education												
0–3 years	09·9	09·1	08·3	09·0	08·1	07·6	07·1	06·8	06·7	06·1	06·1	06·3
4–8 years	33·7	32·9	32·3	29·8	28·6	29·0	28·8	27·8	25·8	24·7	24·2	22·5
9–11 years	34·7	35·8	35·8	36·7	38·5	37·5	38·1	38·1	35·9	37·3	38·0	38·4
≥ 12 years	21·6	22·2	23·5	24·5	24·7	25·9	25·9	27·3	31·6	31·9	31·8	32·8
Sex												
Male	46·1	46·1	46·1	46·1	46·1	46·1	46·1	46·0	46·0	46·0	46·0	46·0
Female	53·9	53·9	53·9	53·9	53·9	53·9	53·9	54·0	54·0	54·0	54·0	54·0
Skin colour/race												
White	39·0	39·2	40·0	43·5	40·6	41·5	39·7	40·8	43·6	42·0	41·3	41·0
Black/Brown	60·0	60·1	59·4	51·2	47·3	46·2	46·5	53·1	46·2	47·9	48·9	50·6
Missing information	00·2	00·2	00·2	01·2	07·4	08·5	09·7	01·7	06·9	07·5	07·6	06·5
Food consumption												
Recommended consumption*												
Fruits and vegetables	20·0	20·2	19·5	22·0	22·7	23·6	24·1	25·2	24·4	23·7	23·1	22·9
Regular consumption†												
Beans‡	65·6	64·9	65·6	67·6	67·5	66·9	66·1	64·8	61·3	59·5	–	59·7
Soft drinks or artificial juice	26·4	26·0	26·8	27·5	26·0	23·3	20·8	19·0	16·5	14·6	14·4	15·0

VIGITEL, Surveillance of Risk and Protective Factors for Chronic Diseases through Telephone Interviews.

* Food consumption of 5 or more servings per day in 5 or more days of the week.

† Food consumption in 5 or more days of the week.

‡ Lack of an available indicator in 2018.

**Fig. 1:**
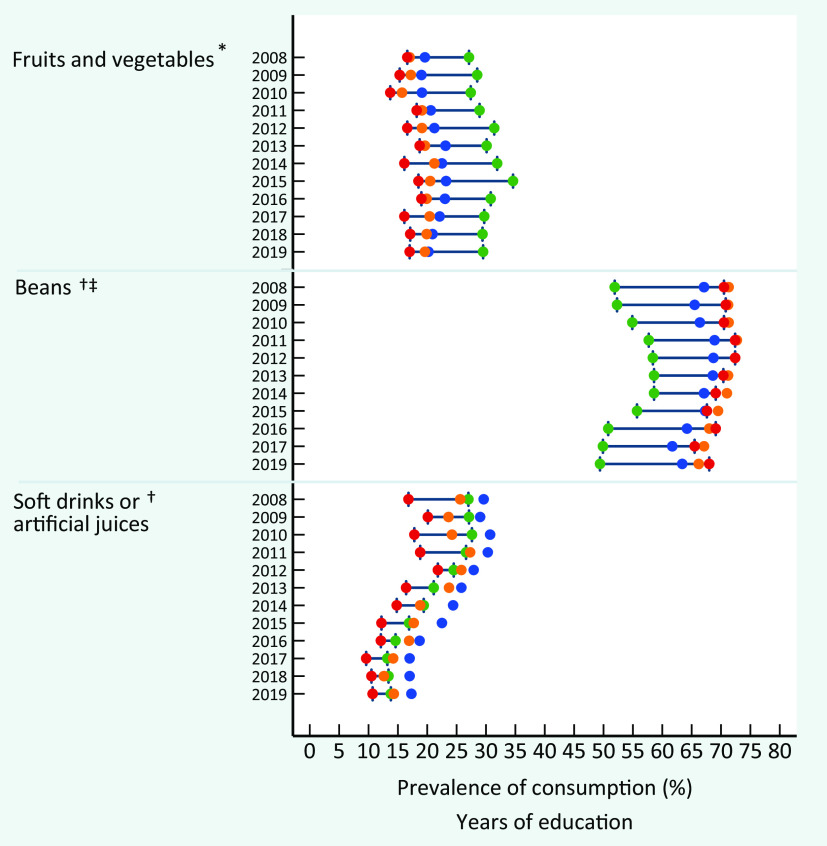
Consumption prevalence of fruits and vegetables, beans and soft drinks or artificial juices in Brazil, by years of education and survey year, VIGITEL 2008–2019 (equiplot). VIGITEL, Surveillance of Risk and Protective Factors for Chronic Diseases through Telephone Interviews. *Food consumption of 5 or more servings per day in 5 or more days of the week. ^†^Food consumption in 5 or more days of the week. ^‡^Lack of an available indicator in 2018. 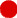, 0–3; 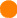, 4–8; 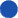, 9–11; 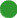, ≥ 12

An important educational gradient was identified for the recommended consumption of fruits and vegetables, characterised by a lower prevalence among individuals with minor schooling levels (Fig. 1). The highest educational inequality was observed for regular consumption of beans, characterised by an expressively lower prevalence in individuals with 12 or more years of education than those with lower schooling levels. On the other hand, the educational discrepancy was small for soft drinks or artificial juices consumption and still presented an expressive long-term reduction among individuals from all educational strata, sharper among individuals with higher schooling levels. A more pronounced frequency of regular consumption of these beverages among individuals with intermediate education (9–11 years of study) is noteworthy.

**Fig. 2:**
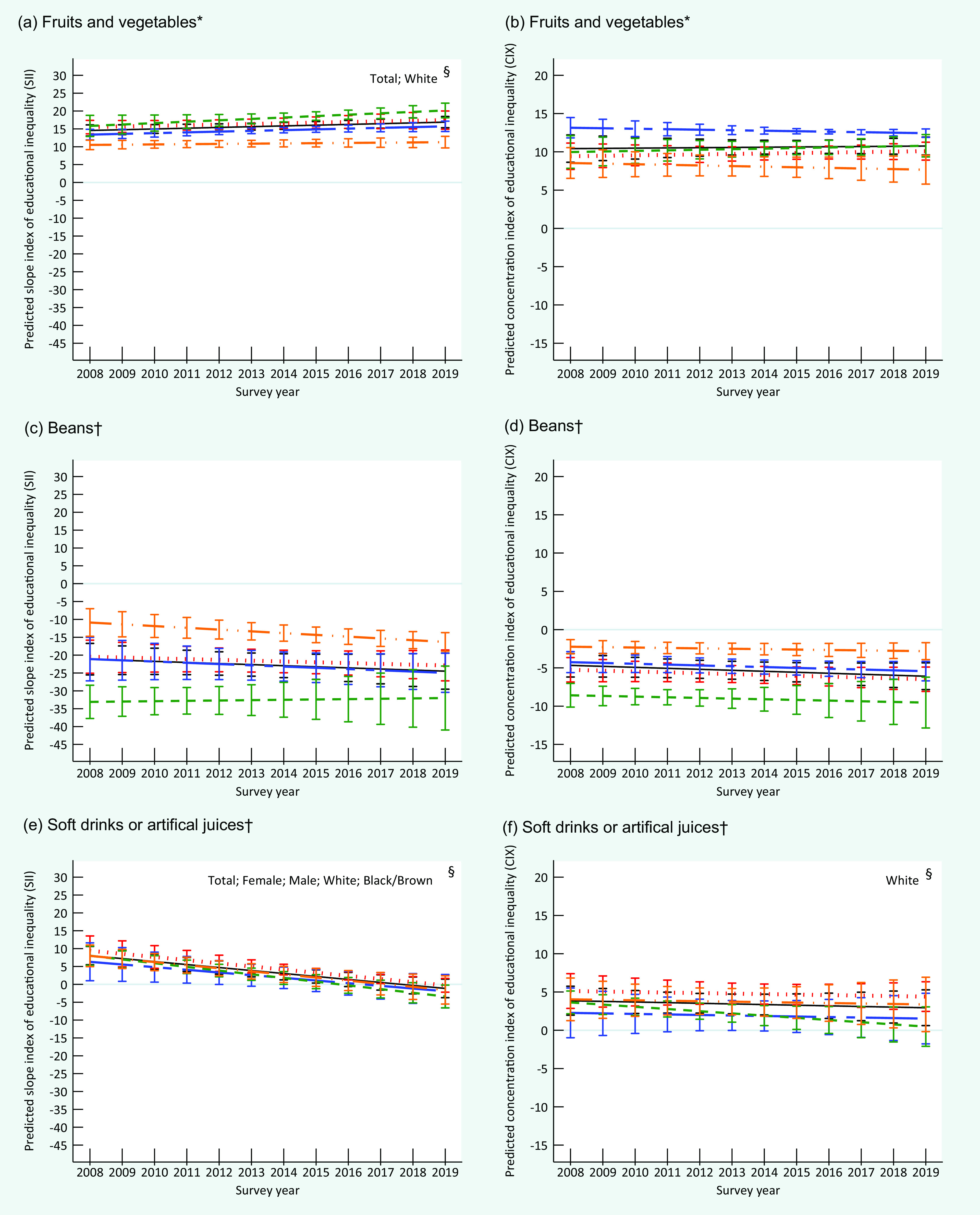
Trends in the predicted slope index of educational inequality (SII) and predicted concentration index of educational inequality (CIX) for consumption prevalence of fruits and vegetables (a; b), beans (c; d) soft drinks or artificial juices (e; f) in Brazil, by sex and skin colour/race, VIGITEL 2008–2019. VIGITEL, Surveillance of Risk and Protective Factors for Chronic Diseases through Telephone Interviews. *Food consumption of 5 or more servings per day in 5 or more days of the week. ^†^Food consumption in 5 or more days of the week. ^§^ *P* < 0·05 (*P*-trend). 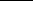, total; 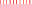, female; 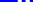, male; 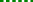, White; 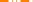, Black/Brown

**Fig. 3:**
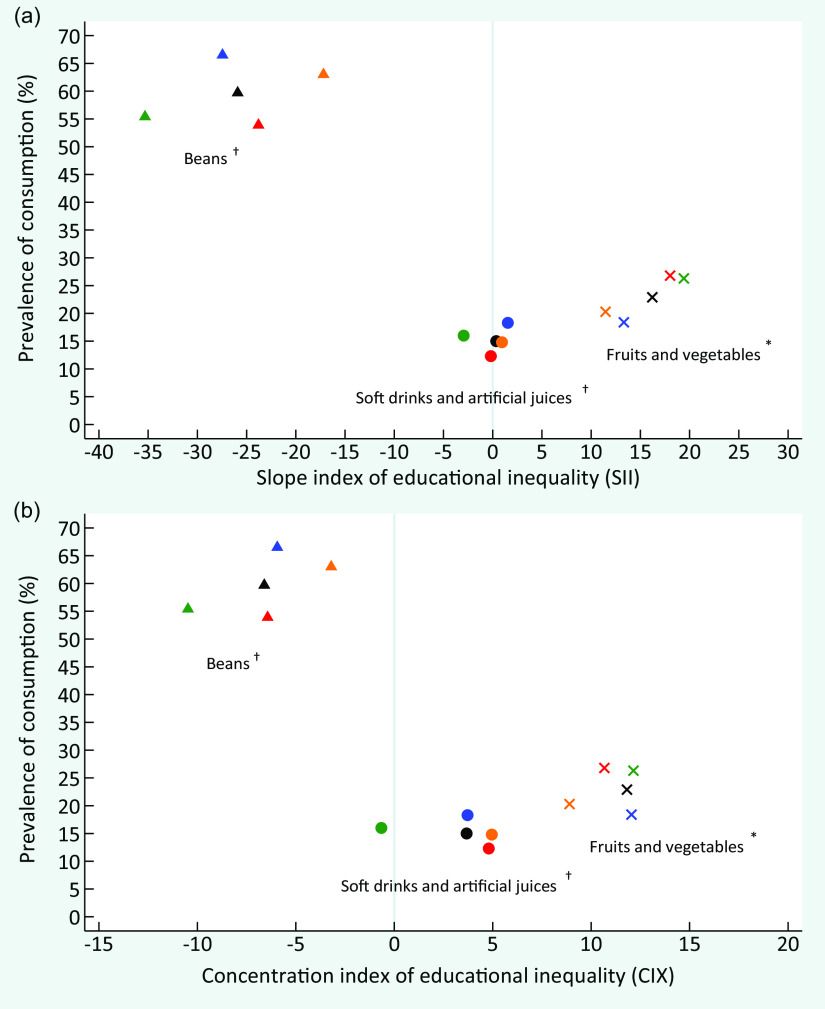
Consumption prevalence of fruits and vegetables, beans and soft drinks or artificial juices in Brazil, by sex and skin colour/race and the slope index of educational inequality (SII) (a) and concentration index of educational inequality (CIX) (b), VIGITEL 2019. VIGITEL, Surveillance of Risk and Protective Factors for Chronic Diseases through Telephone Interviews. *Food consumption of 5 or more servings per day in 5 or more days of the week. ^†^Food consumption in 5 or more days of the week. 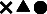, total; 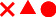, female; 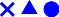, male; 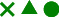, White; 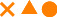, Black/Brown

At the beginning of the period, the absolute and relative educational inequalities for the recommended consumption of fruits and vegetables, represented by positive values of SII and CIX, were similar between men and women and among White and Black/Brown individuals. Over time, there was an increase in absolute educational inequality in the group of all individuals (entire sample) and among White individuals. Relative inequality remained constant for the entire sample and the subgroups analysed over time (Fig. 2). Women and White people presented a higher recommended consumption prevalence of fruits and vegetables in 2019 (Fig. 3).

In contrast, negative values of SII and CIX, representing the respective absolute and relative inequality, were observed for the regular beans’ consumption over the study period. This indicates a higher frequency among less educated individuals. The absolute and relative inequalities were similar between women and men; however, the values were higher among White individuals than in Black/Brown individuals. Absolute and relative inequalities remained constant in all strata throughout the study (Fig. 2). In 2019, educational inequality was higher, and the prevalence of beans regular consumption was lower among White individuals than Black/Brown individuals. Men also presented more elevated prevalence of beans regular consumption than women (Fig. 3).

In 2008, the absolute educational inequality for soft drinks and artificial juices was similar between women and men as well as Whites and Black/Brown individuals, and showed a more frequent consumption among those with high level of education. During the 12 years period, a significant reduction of absolute inequality was identified in all strata, reaching negative SII values among women and White individuals in 2019, indicating that low educated women and White presented higher prevalence of consumption of soft drinks and artificial juices than highly educated ones. The relative inequality remained constant throughout the period in all subgroups, except for White individuals who presented a significant reduction, also attaining a negative CIX in 2019 (Fig. 2). At the end of the period, there was a higher prevalence of regular consumption of these drinks among men, and slightly higher values among White individuals (Fig. 3). We found no relevant relative inequality in whichever food consumption indicator assessed in the present study. Detailed information on characteristics of participants and food consumption according to educational level, sex and skin colour/race is presented in online supplementary material, Supplemental Tables 1–4).

## Discussion

This study has shown that social inequalities in the food consumption of the Brazilian population are manifested as a complex phenomenon. A less predominant recommended consumption of fruits and vegetables among low educated individuals, while regular consumption of beans was less frequent among the more educated. Regular consumption of soft drinks or artificial juices decreased at all schooling levels, between 2008 and 2019, especially among those with more schooling. The absolute educational inequality for the recommended consumption of fruits and vegetables increased in the entire sample and among White individuals. Regular bean consumption was the marker presenting the highest absolute educational inequality, remaining constant throughout the study for all strata. Although the relative education inequality for regular consumption of soft drinks or artificial juices has reduced only among White individuals, the absolute educational inequality reduced in all strata.

Over the period, a slight increase in the prevalence of recommended consumption of fruits and vegetables in Brazilian capitals was identified, reaching in 2015–2016 the target proposed for 2022 (24·3 %) by the Brazilian Strategic Action Plan to Combat Chronic NCD^(16)^. Nevertheless, we observed a gradual reduction from 2017 onwards in the prevalence of recommended consumption below the proposed target (22·9 % in 2019). Considering that family income and food costs are factors influencing the acquisition of fruits and vegetables^(17)^, an intense economic and political crisis in Brazil since 2014 has worsened several social indicators, such as income, unemployment rates, increased food prices^(18,19)^, for example, fruits and vegetables^(20)^ and augmented food insecurity^(18)^. Thus, this crisis could justify the gradual reduction in the consumption of these foods.

Evidence on availability, accessibility and consumption of fruits and vegetables in eighteen countries showed that the cost of two servings of fruit and three servings of vegetables per day per individual requires a substantial proportion of family income, that is, about 52·0 % of family income in low-income countries, 18·0 % and 16·0 % in lower-middle-income countries and upper-middle-income countries, respectively, making this consumption inaccessible in different countries^(21)^. Among those reaching the recommended consumption of fruits and vegetables in the present study, a higher prevalence among more educated individuals was noticed, corroborating previous studies^(2,8,22)^. The highest consumption of fruits and vegetables in the highest income strata was observed in a representative sample of the Brazilian population by the Brazilian Family Budget Surveys, performed in 2017–2018^(23)^.

There was an increase in education inequality for the recommended consumption of fruits and vegetables throughout time. In response to the above-mentioned financial crisis, the Brazilian government adopted austerity measures, such as a budget reduction of an important support and incentive policy for family farming known as the Program for Food Acquisition (PAA)^(19)^. This measure negatively impacts the production of fruits and vegetables and hinders the access to these foods by individuals in the most vulnerable situation^(24)^. Inequality increased among White individuals and remained constant among Black/Brown citizens. This possibly occurred due to persistent racial segregation observed in the Brazilian labour market, which hampers the life conditions of Black and Brown individuals, even the ones with higher education levels, grouping them with their less educated peers. Economic activities resulting in lower average incomes, such as domestic services, construction and agriculture, are proportionally more occupied by Black and Brown individuals, even after adjusting the data according to working hours and schooling^(7)^. Furthermore, the unemployment rate among Black or Brown people has been higher than among White individuals, even when adjusting for educational level^(7)^.

Our outcomes endorse the need to create and strengthen public food policies to promote the availability and equal access to fruits and vegetables through broad and synergistic interventions in the food system, aiming to boost the production, distribution and consumption of these foods and reduce their costs^(21)^ and waste^(25,26)^. Price discounts added to nutritional education activities^(27)^ as well as the presence of food environment with a higher density of healthy food establishments, such as shops specialised in the sales of fruits and vegetables, open markets^(22)^ and community gardens^(28)^, stand out as examples of potential strategies to enhance the current scenario.

Contrasting the recommended consumption of fruits and vegetables, we identified a regular consumption of beans in about half of the individuals, which tended to decline at the end of the period. Family Budget Survey data also brought to light a reduction in the consumption frequency of traditional Brazilian foods, such as beans, between 2008–2009 and 2017–2018 (72·8 % *v*. 60·0 %), albeit it remains one of the most consumed foods in Brazil^(23)^.

Over the study period, there was a noteworthy and constant educational inequality in the regular consumption of beans, characterised by a higher prevalence of this health protector food marker among the less educated, corroborating other studies on less educated people^(4)^ and individuals with lower income^(23)^. Concerning Race/colour, we also identified a significant educational inequality between White and Black/Brown individuals, highlighting that schooling played a key role in determining the differences in the regular consumption of beans among White individuals. The highest consumption of beans has been associated with Black or Brown race/colour in the literature^(4)^, and this profile remained among more educated Black/Brown individuals in our study. Access to basic food, such as beans, is pivotal to promote health and prevent diseases in the population^(4)^.

Beans present a healthy nutritional profile and represent a traditional Brazilian diet^(23)^, usually consumed together with other traditional culinary preparations, including unprocessed or minimally processed foods, such as rice, roots, tubers, maize and other dishes with cereal and eggs^(29)^. Thus, maintaining beans’ regular consumption by the least economically favoured population and encouraging their adherence by the most favoured is of utmost importance.

In these 12 years, we have identified an expressive reduction in the frequency of regular consumption of soft drinks or artificial juices by the population, a more pronounced decrease among the more educated, leading to a significant reduction in educational inequality. A temporal decrease in the consumption of soft drinks and artificial juices was observed in all income classes and, more intensely, in the higher income quarter between 2008–2009 and 2017–2018 in Brazil^(23)^. This aspect is noteworthy since these beverages are considered not healthy^(25)^ and are associated with a higher morbimortality risk^(25,30,31)^.

On the one hand, the Brazilian economic and political crisis impact on food prices and family income could have influenced the reduction in the regular consumption frequency of these beverages among the less educated^(10)^. In contrast, the enhancement of Brazilians education level may have contributed to the decrease in the regular consumption frequency among the most educated, considering that the higher education levels, regardless the income, play a role in healthy food choices^(32)^. The more frequent consumption of these drinks was observed among those with intermediate education and could have been resulted from the association of the greater purchasing power of these foods with insufficient knowledge on the relationship between nutrition and disease^(33)^. Furthermore, the considerable presence of individuals with intermediate schooling in the informal labour market, characterised by arduous activities and/or longer working time^(34)^, is possibly an important limiting factor for healthier food choices, due to physical tiredness and/or limited time available for purchase and preparation of food/drinks at home^(35)^.

In 2019, regular consumption of soft drinks or artificial juices became more frequent among some less educated groups and subgroups of women and White individuals. Bearing in mind the current context, a more consistent and comprehensive inversion of the consumption prevalence of these beverages is potentially predictable among educational strata in the Brazilian population within few years. Thus, a regular consumption profile will tend to prevail among the less socio-economically favoured individuals, which has already been observed more clearly in developed countries^(36,37)^.

Implementing strategies at a population level, such as sweetened drinks taxation, food marketing regulation and nutritional education policies^(38)^, could reduce the consumption of soft drinks or artificial juices among the least favoured individuals and protect them. Estimates point out that a tax designed to increase the retail price of sugary drinks by at least 20·0 % can generate significant changes in consumption habits, especially among vulnerable populations, including low-income consumers, who are more responsive to prices and can benefit more in terms of health. Data from Mexico corroborate this positive taxation impact^(39,40)^. In Brazil, the debate on taxing sweetened beverages persists within the Brazilian tax reform proposal^(41)^, although sweetened beverage companies are paradoxically receiving tax reductions and tax exemptions^(42)^.

The present study reports the most vulnerable groups for the lowest consumption of fruits and vegetables, as well as beans, and the highest consumption of soft drinks or artificial juices. Recent evidence from 195 countries, including data from 1990 to 2017, showed that diets low in fruit, low in vegetables and high in Na (often found in sweetened beverages)^(43)^ are among the main dietary risk factors for mortality, with each factor accounting for more than 2·0 % of global deaths^(25)^. Thus, promoting universal and equitable access to healthy foods and reducing consumption of unhealthy foods, to avoid deaths attributable to dietary risk factors, can be a global response to tackle inequalities and promote a socially and environmentally sustainable food system^(44,45)^. Brazil is an upper-middle-income country^(46)^, and the social and racial inequalities found in food consumption can be used to guide policy makers and nutrition policies to promote healthier diets in Brazil and countries with similar economic and cultural characteristics in Latin America or other continents. Social inequalities in food consumption are a global issue, and even though these countries might have different magnitudes of inequalities, they sure face inequalities challenges like Brazil.

This study’s strengths include the expressive sample size, the use of complex inequality measures, to analyse the extent of social inequality in food consumption, measured by educational gradients, and verify how social inequality changed over 12 years in an upper-middle-income country. Thus, our study provides evidence of the gap trend in food consumption over time, and it calls for further studies in other countries. Furthermore, educational inequality analyses, stratified by sex and ethnicity, were performed due to their relevant inequality dimensions that overlap and can interact with socio-economic differences^(6)^.

However, our study also has some limitations. The VIGITEL sample includes individuals residing in Brazilian capitals and the Federal District with access to landlines, extrapolating that data through weighting measures to obtain representative data of this population. Nevertheless, some differences are expected in the prevalence of food consumption indicators^(47)^, since the most socio-economically favoured Brazilian families are more likely to have a telephone landline^(48)^, and the access to those has decreased since 2015^(49)^. Thus, interviewees could have a higher socio-economic level than the general population, especially in this period, albeit a significant social inequality was observed in food consumption. Due to the low representativeness, we did not include the data of those who self-declared to be Yellow and Indigenous in sub-analysis of skin colour and race. Despite this, our study innovates and advances in the discussion about racial or skin colour inequalities in food consumption in Brazil, showing its trend in a 12-year period between Whites and Blacks/Browns. Only three food groups were evaluated in the study, due to modifications that were made to the VIGITEL questionnaire over time. However, the groups correspond to markers of a healthy (fruits and vegetables, and beans) and unhealthy diet (soft drinks or artificial juices). Furthermore, the information collected by VIGITEL is subject to self-reported classification errors, but it worth mentioning the reliable reproducibility and adequate validity of food and beverage consumption indicators obtained through a surveillance system based on telephone surveys^(50)^. Minute inaccuracies are also identified throughout the consumption evaluation; however, they remain over the entire study period without impacting on the temporal nature outcomes of this study.

In conclusion, the inequality challenge in the recommended consumption of fruits and vegetables has increased in these 12 years, leaving groups with less education more vulnerable. In contrast, the inequality in regular consumption of beans has remained, protecting these groups. Among the subgroups, the absolute inequality for fruits and vegetables increased among White individuals, and the values remained the same for beans and significantly decreased for soft drinks or artificial juices in all analysed strata, albeit the relative inequality diminished only among White individuals. Even though the assessed data for the present study were up to 2019, and not until 2020, the health and economic crisis resulted from COVID-19 pandemic in 2020 will play a detrimental role in Brazilian markets, since it has already impacted food costs, including basic foods in the Brazilian diet^(51)^. Therefore, at this moment, significant and perennial investments in public policies are essential and even more urgent to promote the education of the population, as well subsidise as adequate access and consumption of fruits and vegetables, as well as beans and other legumes, and tackling the excessive consumption of soft drinks or artificial juices.

## Supplementary material

For supplementary material accompanying this paper visit https://doi.org/10.1017/S1368980021002950. click here to view supplementary material
