# Supplementary material for: Social inequality in food consumption between 2008 and 2019 in Brazil
Source: Public Health Nutr. 2021 Aug 19;25(2):214–24. doi: 10.1017/S1368980021002950 (PMC8883783; doi:10.1017/S1368980021002950)
Supplement: Supplementary file 1 [file S1368980021002950sup.zip › S1368980021002950sup002.docx]

**Supplementary Table 1:** Sociodemographic characteristics according to years of education, sex, skin color/race, and survey year in Brazil, VIGITEL 2008-2019.

| Years of education | | | Survey year | | | | | | | | | | | |
| --- | --- | --- | --- | --- | --- | --- | --- | --- | --- | --- | --- | --- | --- | --- |
|  | Sex and skin color / race | | 2008 | 2009 | 2010 | 2011 | 2012 | 2013 | 2014 | 2015 | 2016 | 2017 | 2018 | 2019 |
| 0-3 years | | |  |  |  |  |  |  |  |  |  |  |  |  |
|  | Sex, % | |  |  |  |  |  |  |  |  |  |  |  |  |
|  | | Male | 42.0 | 45.5 | 46.0 | 42.9 | 42.4 | 44.5 | 45.5 | 43.4 | 41.7 | 42.4 | 46.4 | 43.6 |
|  | | Female | 58.0 | 54.5 | 54.0 | 57.1 | 57.6 | 55.5 | 54.5 | 56.6 | 58.3 | 57.6 | 53.6 | 56.4 |
|  | Skin color / race, % | |  |  |  |  |  |  |  |  |  |  |  |  |
|  | | White | 29.1 | 28.3 | 29.3 | 34.4 | 33.3 | 33.6 | 32.5 | 28.9 | 36.1 | 32.7 | 29.8 | 33.6 |
|  | | Black/Brown | 70.0 | 70.9 | 70.0 | 58.5 | 43.1 | 42.2 | 40.3 | 61.6 | 37.3 | 39.1 | 42.9 | 44.7 |
|  | | Missing information | 00.2 | 00.6 | 00.5 | 02.7 | 17.6 | 20.3 | 22.5 | 04.9 | 22.0 | 25.0 | 24.3 | 19.4 |
| 4-8 years | | |  |  |  |  |  |  |  |  |  |  |  |  |
|  | Sex, % | |  |  |  |  |  |  |  |  |  |  |  |  |
|  | | Male | 47.6 | 46.5 | 46.6 | 47.5 | 47.9 | 47.3 | 46.8 | 46.9 | 47.4 | 46.7 | 46.1 | 46.8 |
|  | | Female | 52.4 | 53.5 | 53.4 | 52.5 | 52.1 | 52.7 | 53.2 | 53.1 | 52.6 | 53.3 | 53.9 | 53.2 |
|  | Skin color / race, % | |  |  |  |  |  |  |  |  |  |  |  |  |
|  | | White | 31.7 | 32.1 | 32.8 | 38.2 | 33.9 | 35.5 | 33.0 | 34.0 | 38.7 | 38.1 | 35.3 | 36.1 |
|  | | Black/Brown | 67.5 | 67.3 | 66.6 | 55.8 | 49.3 | 47.2 | 46.9 | 59.5 | 46.0 | 46.6 | 50.3 | 50.6 |
|  | | Missing information | 00.3 | 00.1 | 00.1 | 01.5 | 11.5 | 13.3 | 16.2 | 02.4 | 11.7 | 12.6 | 12.1 | 11.4 |
| 9-11 years | | |  |  |  |  |  |  |  |  |  |  |  |  |
|  | Sex, % | |  |  |  |  |  |  |  |  |  |  |  |  |
|  | | Male | 45.9 | 46.2 | 46.0 | 46.5 | 46.4 | 46.9 | 46.4 | 47.0 | 47.6 | 47.9 | 47.0 | 47.8 |
|  | | Female | 54.1 | 53.8 | 54.0 | 53.5 | 53.6 | 53.1 | 53.6 | 53.0 | 52.4 | 52.1 | 53.0 | 52.2 |
|  | Skin color / race, % | |  |  |  |  |  |  |  |  |  |  |  |  |
|  | | White | 37.4 | 37.4 | 36.8 | 38.1 | 36.8 | 37.8 | 36.1 | 36.4 | 38.3 | 36.0 | 36.4 | 34.3 |
|  | | Black/Brown | 61.9 | 62.1 | 62.9 | 56.8 | 53.3 | 52.1 | 52.5 | 57.3 | 54.0 | 56.2 | 55.9 | 58.8 |
|  | | Missing information | 00.2 | 00.2 | 00.1 | 01.0 | 05.3 | 06.5 | 07.0 | 01.2 | 04.8 | 05.4 | 05.7 | 04.8 |
| ≥ 12 years | | |  |  |  |  |  |  |  |  |  |  |  |  |
|  | Sex, % | |  |  |  |  |  |  |  |  |  |  |  |  |
|  | | Male | 46.1 | 45.7 | 45.5 | 44.9 | 44.6 | 43.9 | 44.9 | 44.4 | 44.1 | 44.0 | 44.6 | 43.7 |
|  | | Female | 53.9 | 54.3 | 54.5 | 55.1 | 55.4 | 56.1 | 55.1 | 55.6 | 55.9 | 56.0 | 55.4 | 56.3 |
|  | Skin color / race, % | |  |  |  |  |  |  |  |  |  |  |  |  |
|  | | White | 57.6 | 57.1 | 58.5 | 61.3 | 56.7 | 55.7 | 54.6 | 56.8 | 55.3 | 54.0 | 54.0 | 53.5 |
|  | | Black/Brown | 40.8 | 41.7 | 40.4 | 34.5 | 36.8 | 37.8 | 38.7 | 38.6 | 39.6 | 40.9 | 40.7 | 42.1 |
|  | | Missing information | 00.2 | 00.3 | 00.3 | 00.5 | 02.7 | 02.7 | 02.7 | 01.0 | 02.0 | 02.7 | 03.1 | 02.6 |

VIGITEL, Surveillance of Risk and Protective Factors for Chronic Diseases through Telephone Interviews.

**Supplementary Table 2:** Recommended consumption prevalence of fruits and vegetables by years of education, sex, skin color, and survey year in Brazil, VIGITEL 2008-2019.

| Years of education | | | Fruits and vegetables* %  Survey year | | | | | | | | | | | |
| --- | --- | --- | --- | --- | --- | --- | --- | --- | --- | --- | --- | --- | --- | --- |
|  | Sex and skin color / race | | 2008 | 2009 | 2010 | 2011 | 2012 | 2013 | 2014 | 2015 | 2016 | 2017 | 2018 | 2019 |
| 0-3 years | | |  |  |  |  |  |  |  |  |  |  |  |  |
|  | Sex, % | |  |  |  |  |  |  |  |  |  |  |  |  |
|  | | Male | 14.7 | 11.5 | 11.4 | 15.9 | 10.7 | 15.5 | 10.9 | 16.0 | 13.3 | 11.3 | 13.1 | 13.9 |
|  | | Female | 17.9 | 18.4 | 15.7 | 19.9 | 20.9 | 21.2 | 20.5 | 20.4 | 23.0 | 19.6 | 20.6 | 19.3 |
|  | Skin color / race, % | |  |  |  |  |  |  |  |  |  |  |  |  |
|  | | White | 19.9 | 16.5 | 14.3 | 18.4 | 20.2 | 18.5 | 17.9 | 20.1 | 20.3 | 16.8 | 19.1 | 15.1 |
|  | | Black/Brown | 15.0 | 14.9 | 13.4 | 17.8 | 13.8 | 18.7 | 13.2 | 18.2 | 17.9 | 15.7 | 16.4 | 19.5 |
| 4-8 years | | |  |  |  |  |  |  |  |  |  |  |  |  |
|  | Sex, % | |  |  |  |  |  |  |  |  |  |  |  |  |
|  | | Male | 11.8 | 13.0 | 12.9 | 14.7 | 14.0 | 15.2 | 16.0 | 17.7 | 15.0 | 14.7 | 14.4 | 15.5 |
|  | | Female | 21.8 | 20.8 | 18.2 | 23.1 | 23.9 | 23.6 | 25.8 | 23.0 | 24.3 | 25.4 | 24.5 | 23.3 |
|  | Skin color / race, % | |  |  |  |  |  |  |  |  |  |  |  |  |
|  | | White | 20.1 | 19.1 | 17.6 | 22.0 | 21.0 | 22.1 | 24.6 | 23.0 | 21.6 | 22.8 | 21.7 | 24.0 |
|  | | Black/Brown | 15.6 | 16.1 | 14.8 | 17.2 | 18.2 | 18.6 | 19.7 | 19.3 | 18.4 | 19.1 | 19.4 | 16.7 |
| 9-11 years | | |  |  |  |  |  |  |  |  |  |  |  |  |
|  | Sex, % | |  |  |  |  |  |  |  |  |  |  |  |  |
|  | | Male | 16.0 | 14.7 | 15.3 | 16.0 | 16.5 | 19.4 | 19.0 | 18.6 | 18.7 | 17.2 | 16.8 | 16.4 |
|  | | Female | 22.6 | 22.6 | 22.3 | 24.6 | 25.2 | 26.4 | 25.5 | 27.3 | 27.0 | 26.7 | 24.6 | 23.7 |
|  | Skin color / race, % | |  |  |  |  |  |  |  |  |  |  |  |  |
|  | | White | 21.2 | 20.9 | 21.7 | 23.6 | 24.3 | 26.7 | 24.6 | 25.7 | 25.4 | 25.4 | 24.0 | 21.3 |
|  | | Black/Brown | 18.5 | 17.8 | 17.6 | 18.9 | 18.9 | 20.5 | 21.4 | 21.2 | 20.8 | 20.0 | 19.2 | 19.1 |
| ≥ 12 years | | |  |  |  |  |  |  |  |  |  |  |  |  |
|  | Sex, % | |  |  |  |  |  |  |  |  |  |  |  |  |
|  | | Male | 22.2 | 23.6 | 23.2 | 23.9 | 25.9 | 25.3 | 26.0 | 29.3 | 25.4 | 24.5 | 24.5 | 23.9 |
|  | | Female | 31.2 | 32.5 | 31.0 | 32.9 | 35.9 | 33.9 | 36.7 | 38.9 | 35.1 | 33.7 | 33.3 | 33.8 |
|  | Skin color / race, % | |  |  |  |  |  |  |  |  |  |  |  |  |
|  | | White | 27.8 | 30.7 | 29.0 | 31.0 | 34.5 | 33.3 | 34.5 | 37.3 | 32.9 | 32.3 | 32.7 | 32.4 |
|  | | Black/Brown | 25.5 | 25.0 | 24.8 | 25.5 | 26.0 | 25.1 | 27.8 | 30.4 | 27.3 | 26.3 | 25.3 | 25.6 |

VIGITEL, Surveillance of Risk and Protective Factors for Chronic Diseases through Telephone Interviews.

^*^ Food consumption of 5 or more servings per day in 5 or more days of the week.

**Supplementary Table 3:** Regular consumption prevalence of beans by years of education, sex, skin color, and survey year in Brazil, VIGITEL 2008-2019.

| Years of education | | | Beans^† ‡^ %  Survey year | | | | | | | | | | | |
| --- | --- | --- | --- | --- | --- | --- | --- | --- | --- | --- | --- | --- | --- | --- |
|  | Sex and skin color / race | | 2008 | 2009 | 2010 | 2011 | 2012 | 2013 | 2014 | 2015 | 2016 | 2017 | 2018 | 2019 |
| 0-3 years | | |  |  |  |  |  |  |  |  |  |  |  |  |
|  | Sex, % | |  |  |  |  |  |  |  |  |  |  |  |  |
|  | | Male | 76.4 | 78.8 | 77.0 | 79.3 | 78.5 | 74.7 | 73.5 | 73.3 | 74.7 | 74.1 | - | 74.6 |
|  | | Female | 66.2 | 64.1 | 65.1 | 67.2 | 67.9 | 67.0 | 65.4 | 63.2 | 65.2 | 59.2 | - | 62.8 |
|  | Skin color / race, % | |  |  |  |  |  |  |  |  |  |  |  |  |
|  | | White | 72.4 | 68.6 | 67.3 | 74.6 | 72.4 | 71.6 | 66.5 | 58.2 | 68.9 | 65.0 | - | 68.5 |
|  | | Black/Brown | 69.8 | 71.7 | 71.9 | 71.1 | 71.3 | 70.8 | 75.3 | 71.3 | 69.4 | 66.7 | - | 67.9 |
| 4-8 years | | |  |  |  |  |  |  |  |  |  |  |  |  |
|  | Sex, % | |  |  |  |  |  |  |  |  |  |  |  |  |
|  | | Male | 78.3 | 77.6 | 76.3 | 79.7 | 79.6 | 77.3 | 77.7 | 74.7 | 75.6 | 73.1 | - | 73.4 |
|  | | Female | 64.9 | 65.6 | 67.0 | 66.4 | 65.8 | 65.7 | 65.1 | 64.8 | 61.1 | 61.8 | - | 59.9 |
|  | Skin color / race, % | |  |  |  |  |  |  |  |  |  |  |  |  |
|  | | White | 70.0 | 69.7 | 69.8 | 71.1 | 70.6 | 69.5 | 69.2 | 68.0 | 65.2 | 65.8 | - | 64.4 |
|  | | Black/Brown | 72.0 | 71.9 | 72.1 | 74.3 | 74.1 | 73.1 | 72.4 | 71.1 | 69.7 | 68.0 | - | 67.9 |
| 9-11 years | | |  |  |  |  |  |  |  |  |  |  |  |  |
|  | Sex, % | |  |  |  |  |  |  |  |  |  |  |  |  |
|  | | Male | 75.9 | 72.5 | 72.9 | 75.0 | 75.6 | 74.4 | 74.0 | 76.2 | 69.5 | 68.7 | - | 70.3 |
|  | | Female | 59.7 | 59.5 | 60.9 | 63.5 | 62.7 | 63.4 | 61.1 | 59.5 | 59.5 | 55.3 | - | 57.1 |
|  | Skin color / race, % | |  |  |  |  |  |  |  |  |  |  |  |  |
|  | | White | 63.6 | 61.8 | 63.9 | 66.5 | 66.6 | 67.6 | 64.1 | 66.6 | 62.0 | 58.2 | - | 62.5 |
|  | | Black/Brown | 69.3 | 67.9 | 67.8 | 70.4 | 70.4 | 70.3 | 68.9 | 68.3 | 65.8 | 63.7 | - | 64.5 |
| ≥ 12 years | | |  |  |  |  |  |  |  |  |  |  |  |  |
|  | Sex, % | |  |  |  |  |  |  |  |  |  |  |  |  |
|  | | Male | 57.0 | 58.6 | 61.4 | 62.6 | 64.0 | 65.1 | 64.8 | 60.4 | 57.8 | 56.4 | - | 55.0 |
|  | | Female | 47.5 | 47.0 | 49.4 | 53.7 | 53.8 | 53.6 | 53.6 | 51.9 | 45.3 | 44.8 | - | 45.0 |
|  | Skin color / race, % | |  |  |  |  |  |  |  |  |  |  |  |  |
|  | | White | 46.6 | 49.4 | 50.4 | 55.0 | 55.0 | 54.6 | 54.5 | 51.3 | 46.2 | 45.8 | - | 44.3 |
|  | | Black/Brown | 59.5 | 56.6 | 61.3 | 62.6 | 65.0 | 65.2 | 66.0 | 62.1 | 57.6 | 55.8 | - | 55.4 |

VIGITEL, Surveillance of Risk and Protective Factors for Chronic Diseases through Telephone Interviews.

^†^ Food consumption in 5 or more days of the week.

^‡^ Lack of an available indicator in 2018.

**Supplementary Table 4:** Regular consumption prevalence of soft drinks or artificial juices by years of education, sex, skin color, and survey year in Brazil, VIGITEL 2008-2019.

| Years of education | | | Soft drinks or artificial juices^†^ %  Survey year | | | | | | | | | | | |
| --- | --- | --- | --- | --- | --- | --- | --- | --- | --- | --- | --- | --- | --- | --- |
|  | Sex and skin color / race | | 2008 | 2009 | 2010 | 2011 | 2012 | 2013 | 2014 | 2015 | 2016 | 2017 | 2018 | 2019 |
| 0-3 years | | |  |  |  |  |  |  |  |  |  |  |  |  |
|  | Sex, % | |  |  |  |  |  |  |  |  |  |  |  |  |
|  | | Male | 19.7 | 25.7 | 21.2 | 23.1 | 27.0 | 20.8 | 16.3 | 12.1 | 15.8 | 11.3 | 14.0 | 12.9 |
|  | | Female | 14.7 | 15.3 | 14.9 | 15.5 | 18.0 | 12.9 | 13.6 | 12.2 | 09.4 | 08.3 | 07.4 | 09.1 |
|  | Skin color / race, % | |  |  |  |  |  |  |  |  |  |  |  |  |
|  | | White | 17.7 | 20.5 | 18.7 | 19.4 | 22.2 | 15.3 | 15.7 | 13.1 | 12.8 | 10.4 | 08.4 | 12.1 |
|  | | Black/Brown | 16.5 | 19.8 | 17.4 | 18.4 | 23.1 | 18.0 | 17.3 | 11.0 | 12.8 | 10.8 | 08.4 | 09.6 |
| 4-8 years | | |  |  |  |  |  |  |  |  |  |  |  |  |
|  | Sex, % | |  |  |  |  |  |  |  |  |  |  |  |  |
|  | | Male | 28.9 | 27.7 | 27.7 | 33.2 | 31.8 | 28.2 | 21.0 | 21.0 | 22.1 | 17.9 | 15.8 | 17.6 |
|  | | Female | 22.6 | 20.0 | 21.2 | 22.0 | 20.2 | 19.6 | 17.0 | 14.9 | 12.2 | 10.9 | 09.9 | 11.3 |
|  | Skin color / race, % | |  |  |  |  |  |  |  |  |  |  |  |  |
|  | | White | 25.3 | 23.1 | 22.7 | 25.4 | 24.2 | 22.3 | 17.4 | 18.5 | 16.5 | 15.4 | 11.3 | 16.4 |
|  | | Black/Brown | 25.8 | 23.9 | 25.1 | 28.7 | 25.8 | 26.1 | 20.4 | 17.5 | 18.2 | 14.2 | 13.4 | 13.7 |
| 9-11 years | | |  |  |  |  |  |  |  |  |  |  |  |  |
|  | Sex, % | |  |  |  |  |  |  |  |  |  |  |  |  |
|  | | Male | 34.6 | 32.2 | 34.4 | 33.3 | 30.4 | 29.4 | 27.9 | 26.8 | 20.9 | 19.8 | 20.5 | 20.1 |
|  | | Female | 25.3 | 26.3 | 27.4 | 27.7 | 25.8 | 22.7 | 21.4 | 18.7 | 16.7 | 14.4 | 13.8 | 14.7 |
|  | Skin color / race, % | |  |  |  |  |  |  |  |  |  |  |  |  |
|  | | White | 30.0 | 28.8 | 30.2 | 31.9 | 26.9 | 25.0 | 24.9 | 24.2 | 18.6 | 18.6 | 16.6 | 18.6 |
|  | | Black/Brown | 29.4 | 29.0 | 30.9 | 29.3 | 28.8 | 26.8 | 24.6 | 21.9 | 19.2 | 16.2 | 17.2 | 16.9 |
| ≥ 12 years | | |  |  |  |  |  |  |  |  |  |  |  |  |
|  | Sex, % | |  |  |  |  |  |  |  |  |  |  |  |  |
|  | | Male | 31.7 | 28.5 | 29.4 | 31.7 | 27.2 | 22.4 | 23.3 | 19.7 | 16.5 | 15.2 | 16.3 | 17.5 |
|  | | Female | 22.9 | 25.9 | 26.2 | 22.5 | 22.3 | 20.2 | 16.2 | 14.7 | 13.0 | 11.6 | 11.0 | 10.9 |
|  | Skin color / race, % | |  |  |  |  |  |  |  |  |  |  |  |  |
|  | | White | 28.1 | 27.6 | 28.8 | 27.4 | 24.2 | 20.5 | 19.9 | 17.0 | 14.6 | 13.9 | 13.2 | 14.3 |
|  | | Black/Brown | 26.0 | 26.4 | 26.1 | 25.2 | 25.2 | 21.8 | 18.4 | 16.9 | 14.8 | 12.4 | 13.8 | 13.4 |

VIGITEL, Surveillance of Risk and Protective Factors for Chronic Diseases through Telephone Interviews.

^†^ Food consumption in 5 or more days of the week.
