# Supplementary material for: Social inequality in food consumption between 2008 and 2019 in Brazil
Source: Public Health Nutr. 2021 Aug 19;25(2):214–24. doi: 10.1017/S1368980021002950 (PMC8883783; doi:10.1017/S1368980021002950)
Supplement: Supplementary file 1 [file S1368980021002950sup.zip › S1368980021002950sup001.doc]

STROBE Statement—Checklist of items that should be included in reports of ***cross-sectional studies***

|  | Item No | Recommendation |
| --- | --- | --- |
| **Title and abstract** | 1 | Indicate the study’s design with a commonly used term in the title or the abstract  Page 1 |
|  |  | (*b*) Provide in the abstract an informative and balanced summary of what was done and what was found Page 1 |
| Introduction | | |
| Background/rationale | 2 | Explain the scientific background and rationale for the investigation being reported  Page 2 |
| Objectives | 3 | State specific objectives, including any prespecified hypotheses  Pages 2-3 |
| Methods | | |
| Study design | 4 | Present key elements of study design early in the paper  Page 3 |
| Setting | 5 | Describe the setting, locations, and relevant dates, including periods of recruitment, exposure, follow-up, and data collection  Page 3 |
| Participants | 6 | Give the eligibility criteria, and the sources and methods of selection of participants  Page 3 |
| Variables | 7 | Clearly define all outcomes, exposures, predictors, potential confounders, and effect modifiers. Give diagnostic criteria, if applicable  Pages 3-4 |
| Data sources/ measurement | 8* | For each variable of interest, give sources of data and details of methods of assessment (measurement). Describe comparability of assessment methods if there is more than one group Pages 4-5 |
| Bias | 9 | Describe any efforts to address potential sources of bias Pages 4-5 |
| Study size | 10 | Explain how the study size was arrived at Page 3 |
| Quantitative variables | 11 | Explain how quantitative variables were handled in the analyses. If applicable, describe which groupings were chosen and why Page 4 |
| Statistical methods | 12 | (*a*) Describe all statistical methods, including those used to control for confounding Page 5 |
|  |  | (*b*) Describe any methods used to examine subgroups and interactions Pages 4-5 |
|  |  | (*c*) Explain how missing data were addressed Page 3 |
|  |  | (*d*) If applicable, describe analytical methods taking account of sampling strategy Page 5 |
|  |  | (*e*) Describe any sensitivity analyses NA |
| Results | | |
| Participants | 13* | (a) Report numbers of individuals at each stage of study—eg numbers potentially eligible, examined for eligibility, confirmed eligible, included in the study, completing follow-up, and analysed Page 3; Table 1 |
|  |  | (b) Give reasons for non-participation at each stage  Page 3; Table 1; Fig. 1; Supplementary Table 3. |
|  |  | (c) Consider use of a flow diagram NA |
| Descriptive data | 14* | (a) Give characteristics of study participants (eg demographic, clinical, social) and information on exposures and potential confounders  Pages 5-6; Table 1; Supplementary Table 1. |
|  |  | (b) Indicate number of participants with missing data for each variable of interest Page 3 |
| Outcome data | 15* | Report numbers of outcome events or summary measures  Table 1; Supplementary Table 2, 3 and 4. |
| Main results | 16 | (*a*) Give unadjusted estimates and, if applicable, confounder-adjusted estimates and their precision (eg, 95% confidence interval). Make clear which confounders were adjusted for and why they were included Figures 1, 2 and 3 |
|  |  | (*b*) Report category boundaries when continuous variables were categorized NA |
|  |  | (*c*) If relevant, consider translating estimates of relative risk into absolute risk for a meaningful time period NA |
| Other analyses | 17 | Report other analyses done—eg analyses of subgroups and interactions, and sensitivity analyses Figures 1, 2 and 3 |
| Discussion | | |
| Key results | 18 | Summarise key results with reference to study objectives Page 7 |
| Limitations | 19 | Discuss limitations of the study, taking into account sources of potential bias or imprecision. Discuss both direction and magnitude of any potential bias  Page 10 |
| Interpretation | 20 | Give a cautious overall interpretation of results considering objectives, limitations, multiplicity of analyses, results from similar studies, and other relevant evidence  Pages 7-10 |
| Generalisability | 21 | Discuss the generalisability (external validity) of the study results Page 10 |
| Other information | | |
| Funding | 22 | Give the source of funding and the role of the funders for the present study and, if applicable, for the original study on which the present article is based NA |

*Give information separately for exposed and unexposed groups.

**Note:** An Explanation and Elaboration article discusses each checklist item and gives methodological background and published examples of transparent reporting. The STROBE checklist is best used in conjunction with this article (freely available on the Web sites of PLoS Medicine at http://www.plosmedicine.org/, Annals of Internal Medicine at http://www.annals.org/, and Epidemiology at http://www.epidem.com/). Information on the STROBE Initiative is available at www.strobe-statement.org.
